# Economic analysis of two-stage septic revision after total hip arthroplasty: What are the relevant costs for the hospital’s orthopedic department?

**DOI:** 10.1186/s12891-016-0962-6

**Published:** 2016-03-01

**Authors:** R. Kasch, G. Assmann, S. Merk, T. Barz, M. Melloh, A. Hofer, H. Merk, S. Flessa

**Affiliations:** Center of Orthopaedics, Trauma Surgery and Rehabilitation Medicine, Clinic and Outpatient Clinic for Orthopaedics and Orthopaedic Surgery, University Medicine Greifswald, Greifswald, Germany; Department of Health Care Management, Faculty of Law and Economics, Ernst-Moritz-Arndt-University, Greifswald, Germany; Department of Orthopedics and Trauma Surgery, Asklepios Hospital Uckermark, Schwedt, Germany; Center for Health Sciences, School of Health Professions, Zurich University of Applied Sciences, Winterthur, Switzerland

**Keywords:** Septic, Revision, Total hip arthroplasty, Cost, Reimbursement, Contribution margin

## Abstract

**Background:**

The number of septic total hip arthroplasty (THA) revisions is increasing continuously, placing a growing financial burden on hospitals. Orthopedic departments performing septic THA revisions have no basis for decision making regarding resource allocation as the costs of this procedure for the departments are unknown. It is widely assumed that septic THA procedures can only be performed at a loss for the department. Therefore, the purpose of this study was to investigate whether this assumption is true by performing a detailed analysis of the costs and revenues for two-stage septic THA revision.

**Methods:**

Patients who underwent revision THA for septic loosening in two sessions from January 2009 through March 2012 were included in this retrospective, consecutive cost study from the orthopedic department’s point of view. We analyzed variable and case-fixed costs for septic revision THA with special regard to implantation and explantation stay. By using marginal costing approach we neglected hospital-fixed costs. Outcome measures include reimbursement and daily contribution margins.

**Results:**

The average direct costs (reimbursement) incurred for septic two-stage revision THA was €10,828 (€24,201). The difference in cost and contribution margins per day was significant (*p* < .001 and *p* = 0.019) for ex- and implantation (€4147 vs. €6680 and €429 vs. €306) while length of stay and reimbursement were comparable.

**Conclusions:**

This is the first detailed analysis of the hospital department’s cost for septic revision THA performed in two sessions. Disregarding hospital-fixed costs the included variable and case fixed-costs were covered by revenues. This study provides cost data, which will be guidance for health care decision makers.

## Background

Currently, one million primary total hip arthroplasties (THA) are performed worldwide each year [1]. It has been estimated that this number will double over the next two decades [1]. Factors contributing to this expected increase include the demographic trend of a rapidly aging population and the fact that today’s elderly people are more active than previous generations. These demographic developments as well as the tendency to perform hip replacement surgery at an increasingly earlier age contribute to a growing number of individuals with artificial hips. Naturally, also the number of patients who need a revision THA due to septic loosening will increase [2–5]. Periprosthetic infection is a devastating complication in patients with hip prosthesis and is associated with higher mortality, morbidity, and health care use [1, 2]. For chronic hip infections, two-stage revision is the gold standard [1, 6]. Septic hip revision is a technically complex and time-consuming procedure and requires more resources than primary THA [1, 4, 6–8]. The duration of hip revision surgery is longer, the prostheses are more expensive, surgical implantation costs are higher, patients stay in hospital longer, and there are more complications [4, 7].

This study was performed in a hospital setting being reimbursed by diagnosis related groups (DRG), which follows the fee-per-case approach. In most health care systems, hospitals are reimbursed on the basis of DRGs rather than on daily rates. In the past it got more and more challenging to ensure that at least their costs are recovered when fulfilling their obligation to treat patients. Hospitals making a deficit may not survive. Thus, hospitals and individual departments must analyze their costs to have a sound basis for decision making. For an orthopedic department, this means that the relevant costs of septic THA need to be calculated for proper resource allocation and decisions about the service portfolio.

With the exception of a few highly specialized centers, most hospitals perform a wide spectrum of different procedures. Ideally, the total revenues accruing to a hospital from this mix of procedures should be high enough to cover all liabilities. For a hospital to operate economically, the reimbursement for complex cases that require high levels of resources should at least cover the variable costs per case, while the revenues from other, more common procedures should pay the hospital’s total fixed costs [7, 9].

### Contribution margin approach

**Fig. 1:**
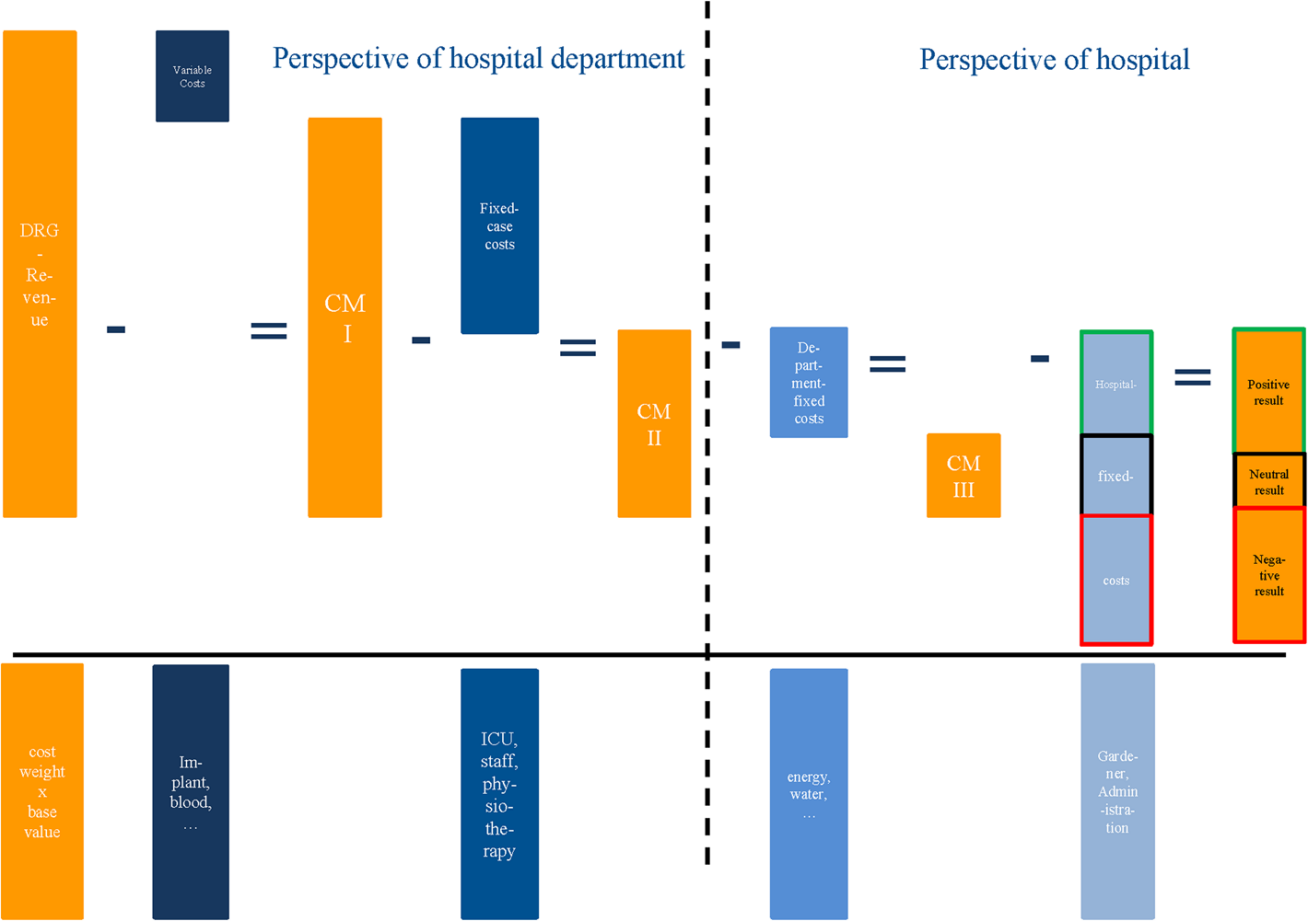
Contribution margin approach (CM = Contribution Margin, DRG = Diagnosis related groups)

Most international studies published in the literature that analyze the costs of septic revision THA use a total cost approach, not separating between fixed and variable costs [7, 10–13]. The total cost approach does not provide data on which a hospital department can base future decisions [9, 14], while the contribution margin approach could provide data that can serve as a basis for decision making. A hospital has variable, fixed case and hospital-fixed costs. In cost accounting, variable costs are expenses that increase or decrease in direct relation to the services or production volume. In the case of a hospital, variable costs are costs that change with the number of patients treated (e.g., costs for implants or medication) [15, 16]. In contrast, the hospital’s fixed costs are independent of how many patients are treated (e.g., costs for energy, gardening, administration). Such fixed costs can only be modified in the long term and are neglected in the contribution margin approach [17]. Fixed case costs, like variable case costs, are assigned to the respective department treating the patients. Unlike variable case costs, fixed case costs are not related to the treatment of an individual patient but simply arise because there are patients with that diagnosis (e.g., personnel costs, operation of an intensive care unit (ICU)). Figure 1 illustrates the marginal costing approach in detail. All costs are illustrated in orange, while reimbursement and contribution margins are colored in blue.

Contribution margin I refers to the difference between net revenues and variable costs. Subtraction of variable and fixed case costs from overall revenues yields contribution margin II. If the result is negative, the orthopedic department incurs a deficit from the treatment of patients with this diagnosis, which must be compensated for by the revenues from other procedures. If the result is positive, no conclusions can be drawn as to whether outcome is also positive for the hospital because hospital-fixed costs (e.g., cost of building maintenance) still have to be considered. The contribution margin approach provides data on the financial outcome for a given hospital department, in our case the orthopedic department. From its internal perspective, the department can make economic, future-oriented decisions without taking into account the hospital’s overall fixed costs.

### Objectives

We analyzed cost structures and contribution margins for septic revision THA procedures performed in two sessions. With this approach, the actual cost associated with a procedure can be calculated, and the orthopedic department performing the procedure can estimate whether the reimbursement for this treatment covers its cost. In addition, we present a detailed analysis of all steps involved in septic revision THA and the corresponding cost separately for the explantation and the implantation procedure. We consider this separate calculation important since, to the best of our knowledge, this has not been done by investigators before. In agreement with most of the published literature, we hypothesize that the relevant variable and fixed case costs associated with septic revision THA are not covered by current reimbursement, resulting in a loss to the hospital [7, 13, 18].

## Methods

### Setting

The analysis was performed using data from a university-affiliated hospital in central Europe providing maximum care. In 2012, 36,345 patients were treated in the entire hospital, which provides 926 beds in 21 departments. In 2012, the Clinic and Outpatient Clinic for Orthopedics and Orthopedic Surgery provided inpatient health care for more than 2000 cases [19].

### Inclusion and exclusion criteria for patient data

Retrospectively, all consecutive patients undergoing revision THA in two sessions for septic loosening from January 2009 to March 2012 were included. The analysis was based on medical and financial data provided by the Department of Medical Controlling. We first identified all inpatients with a supplementary diagnosis of “Septic - infection and inflammatory reaction due to internal joint prosthesis” (ICD-10 Code T84.5) and a positive microbiological test result (positive for bacteria). In a second step, patients not treated according to OPS codes 5–821 (Re-operation, exchange or removal of an artificial hip joint) and 5–820 (Implantation of an artificial hip joint) were eliminated from this initially identified set. The remaining cases were assessed by two experienced hip surgeons who reviewed the surgeons’ reports using a list of predefined eligibility criteria: (i) typical pain in the leg; (ii) imaging evidence of loosening; and (iii) characteristic laboratory and pathophysiologic parameters in blood and biopsy specimens from the joint. This data was obtained from the patient record form including clinical and operation notes as well as lab tests.

### Economic analysis

The reimbursement of healthcare providers on the basis of diagnosis-related groups (DRGs) all over the world means that hospitals receive a fixed payment for a procedure. The amount is calculated on the basis of the average costs of a set of similar procedures [9]. In our analysis, we calculated the reimbursement the hospital received from health insurers using the DRG-associated cost weights from the case fee catalogue as multiplicators and the base value for 2013, which was €3019.9. This amount has to cover all costs, including variable, case-fixed and hospital fixed (=overhead) costs (see Fig. 1). Both hospital stays (explantation and implantation) are reimbursed separately, thus revenues occur for each stay and could be compared with the corresponding costs.

We performed a cost calculation using contribution margins as described previously by Kasch et al. [14].

Costs were analyzed using the parameters listed in Table 1. These parameters were identified and analyzed in terms of frequency during the hospital stay of a patient to then assign the costs they generated. Since hospitals typically negotiate prices for materials with suppliers, we used the hospital’s actual purchase prices for the analysis rather than official list prices (medication, implants, expenditure materials). For calculation of staff costs we measured individual times of involvement, and calculated costs by multiplying the average costs per minute with the times measured. If patients were transferred to the ICU after the operation these costs were included as well. Information on length of ICU stay was available to the minute. The mean orthopedic cost of one ICU day (24 h), we used for calculation, was €1912.3.

**Table 1:** Performance and cost areas for the analyzed cases

Location of cost generation	Cause of costs	Unit	Costs per unit [€]	Cost [€]
Normal ward				
Nursing staff	Nursing staff time	[ppr min]	Costs per min	Time required x cost per unit of time
Laboratory	Laboratory tests	[Points]	Ø Point value	Points x Point value
Radiology	Radiological tests	[Points]	Ø Point value	Points x Point value
Physiotherapy	Physiotherapy	[Points]	Ø Point value	Points x Point value
Medication	Medication during whole stay	[€]	[€]	Direct costs
ICU	ICU treatment	[min]	Costs per min	Time required x costs per unit of time
Surgical costs				
Orthopedics (doctor)	Orthopedic surgeon time	[min]	Costs per min	Time required x costs per unit of time
Orthopedics (nursing)	Surgical nursing staff time	[min]	Costs per min	Time required x costs per unit of time
Anesthesiology (doctor)	Anesthesiologist time	[min]	Costs per min	Time required x costs per unit of time
Anesthesiology (nursing)	Anesthesiological nursing staff time	[min]	Costs per min	Time required x costs per unit of time
Implant	Implant	[€]	[€]	Direct costs
Expendable material	Materials used	[€]	[€]	Direct costs
Sterilization	Sterilization	[sterilization box]	Costs per sterilization box	Number of sterilization boxes x costs per sterilization of one box
Blood products	Blood transfusions	[€]	[€]	Direct costs

*ICU* intensive care unit

Variable costs included cost for implant, drugs, blood transfusions, laboratory, radiology, expenditure material while case-fixed costs remained to staff, physiotherapy and ICU. Since hospitals typically negotiate prices for materials with suppliers, we used the hospital’s actual purchase prices for the analysis rather than official list prices.

For the orthopedic department, the relevant parameter of economic provision of services is the daily contribution margin, which is calculated by dividing average marginal contribution II by average length of hospital stay. The smaller the daily marginal contribution, the more difficult it becomes for the entire hospital to recover its fixed costs. It is essential to know the contribution margin per day of individual DRGs for comparison with other procedures in order to identifying those with the biggest budgetary burden. Table 2 summarizes the most important economic terms used here.

**Table 2:** Definition of economic terms

Term	Explanation	Example
DRG Revenue	Net Revenue for single case	Cost Weight of DRG x Base rate (€3019.9)
- Variable Costs	Costs assigned to a single case	Expendable material, Implants, Sterilization, Radiology, Laboratory, Blood Products, Medication
= Marginal Contribution I		
- Case-Fixed Costs	Costs assigned to a single hospital department	Physician Surgery, Nursing Surgery, ICU, Nursing Staff – Normal Ward, Physiotherapy
= Marginal Contribution II		
- Hospital-Fixed Costs	Costs assigned to the entire hospital	Administrative Personal, Energy, Water, Building Maintenance, Gardening
= Operational Result		
ᅟ		
Perspective of Hospital Department		
Marginal Contribution II/day	Daily contribution of single case to cover hospital-fixed costs	Marginal Contribution II: Average stay of single case

*DRG* diagnosis related groups, *ICU* intensive care unit

In patients undergoing revision THA in two sessions, each of the two hospital stays (removal of the septically-loosened prosthesis and implantation of a new prosthesis) is reimbursed separately and hence we also regard them as two separate stays in our analysis. This is the only possible approach that allows identification of the factors that provide a basis for well-founded economic decisions. The two stays per patient can also be aggregated to calculate total cost for comparison with other procedures or published data.

### Statistical analysis and ethical approval

All data are given as means with ranges. Most cost data are not normally distributed. We tested for normal distribution (Kolmogorow-Smirnow test), and used the nonparametric Mann–Whitney *U*-test for skewed data. Normally distributed data were assessed using the Chi^2^-test and t-test, assuming statistical significance at *p* < .05. All statistical tests were performed using SPSS, version 20. The local ethics committee of Ernst-Moritz-Arndt University Greifswald approved the study (BB 010/13) and waived written informed consent.

## Results

### Patient data

Our search retrieved a total of 357 patients who met the inclusion criteria of an ICD-10 T84.5 supplementary diagnosis. All of them were tested for infection via joint aspiration. A positive microbiological culture was the indication for the periprosthetic infection, knowing that there are also septic joints which are culture negative.

Three-hundred-and-twenty-one cases were eliminated, either because they did not meet the specific eligibility criteria for the medical OPS procedures 5–821 and 5–820 (*n* = 321) or they did not meet eligibility criteria in the surgical reports (*n* = 6). Thirty patients were finally included in our analysis. All of them received a complete THA revision. Further characteristics are summarized in Table 3.

**Table 3:** Baseline demographic and clinical characteristics of the 30 patients included in the analysis of septic revision THA. Continuous data are mean (range), categorical data are counts (%)

	All cases
Total	30
Left [number] (%)	18.0 (60)
Right [number] (%)	12.0 (40)
Age [years], (range)	66.1 (43–81)
Gender	
Male [number] (%)	18 (60.0)
Female [number] (%)	22 (40.0)
Pathogen [number of isolations] (%)	
*Staphylococcus aureus* (all methicillin-sensitive)	19 (63.2)
*Streptococcus agalactiae* (group B Streptococcus)	5 (15.8)
*Coagulase-negative staphylococci*	3 (10.5)
*Enterococcus faecalis*	3 (10.5)
Number of diagnoses (range)	9.3 (4–19)
Number of operations (range)	2.4 (2–5)
Length of stay	40.2 (25–61)
Explantation, [days], (range)	20.5 (13–36)
Implantation, [days], (range)	19.8 (10–33)
Operation time	258.4 (117–557)
Explantation [minutes], (range)	126.5 (52–404)
Implantation [minutes], (range)	131.9 (64–282)

The 30 patients included in our analysis had a mean age of 66.1 years with women having a significantly higher mean age than men (70.6 versus 62.8 years; *p* = 0.048). Most patients (63.2 %) had infection with *Staphylococcus aureus* (all methicillin-sensitive). The total duration of both hospital stays per patient taken together ranged from 25 to 61 days with a mean of 40.2 days. The mean duration of the first hospital stay (explantation) was 20.5 days (13–36), while that of the second stay (implantation) was 19.8 days (10–33) (*p* = 0.982). In the patient records, a mean of 9.3 additional ICD-10 codes were documented; there were no significant differences between men and women (*p* = 0.819).

### Economic data

#### Cost

Table 1 presents the cost items we analyzed separately for explantation and implantation procedures.

The total average costs incurred by the hospital department for each procedure were €4147.4 for explantation and €6680.7 for implantation (*p* < .001). Cost items with significant differences between explantation and implantation were costs for the implant (*p* < .001), sterilization (*p* < .001), normal ward nursing services (*p* < .001), and medication (*p* < .001).

**Fig. 2:**
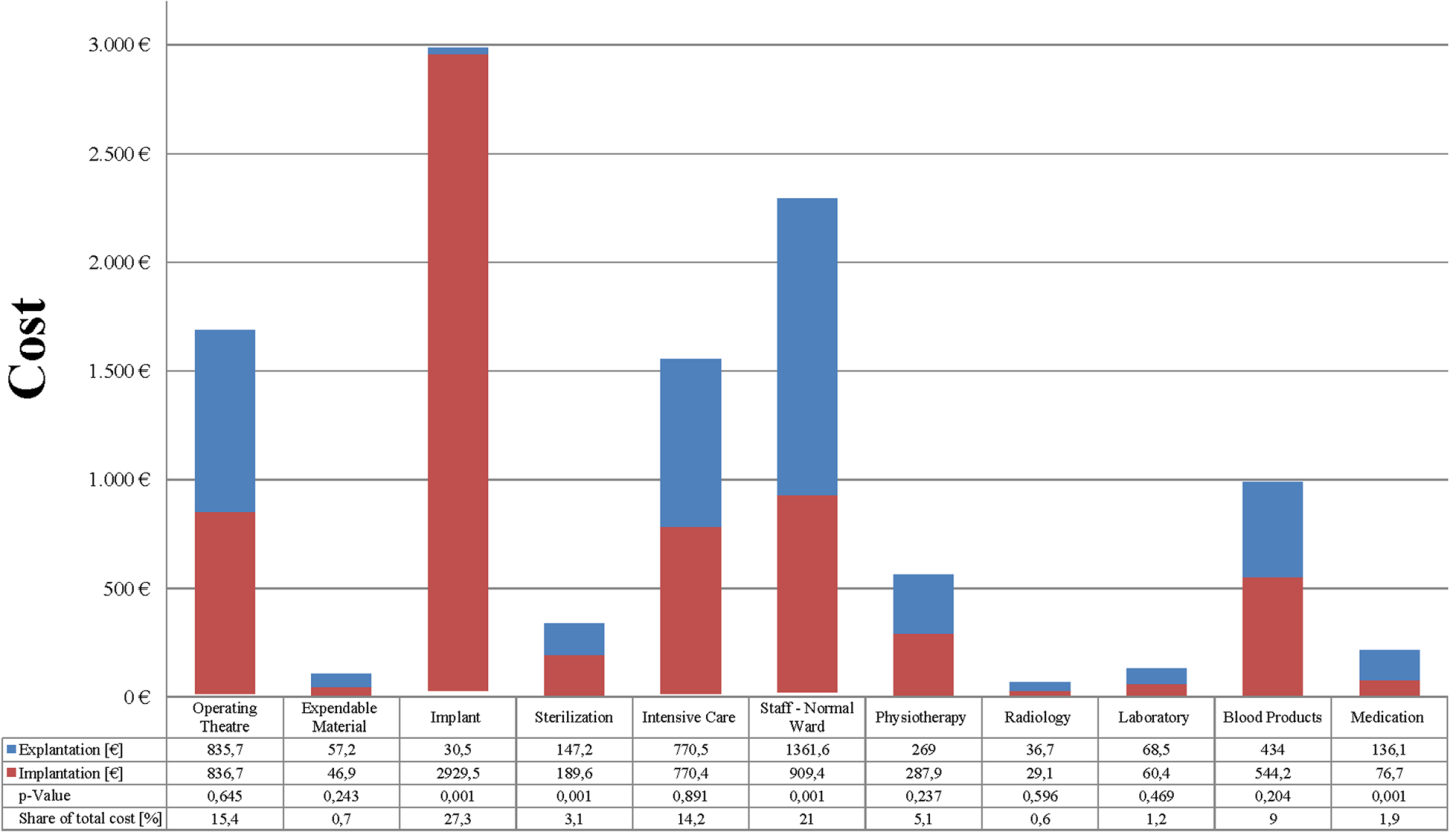
Analysis of explantation and implantation costs for septic revision total hip arthroplasty

For both procedures taken together, the average cost per patient was €10,828.1. The cost listing in Fig. 2 indicates that the largest shares of cost were accounted for by the implant (27.3 %) and salaries for staff in the normal ward (21.0 %), while salaries for the operating theatre were accounted for 15.4 %. The hospital stay for explantation accounted for 38.3 % of the average total cost, while the second hospitalization, for implantation of the new hip endoprosthesis components, accounted for 61.7 %.

**Fig. 3:**
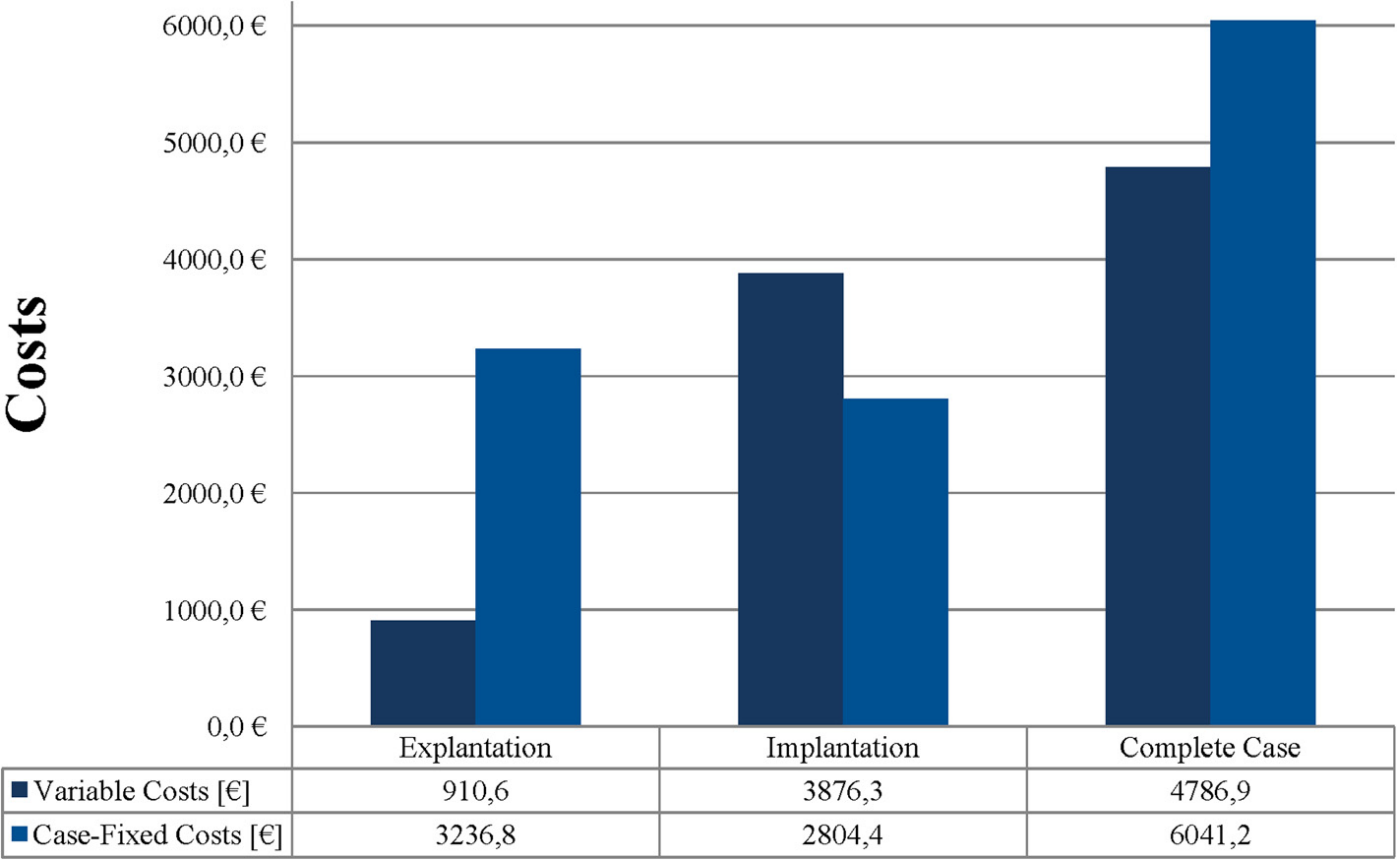
Share of variable and case-fixed costs for explantation, implantation and aggregated total hospital stay of septic revision total hip arthroplasty

Figure 3 presents the relevant variable cost items (implant, blood products, sterilization instruments and materials, expendable materials, laboratory tests, radiologic tests) and fixed case cost items (normal ward staff costs, ICU, surgery staff costs, physiotherapy) for first hospital stay (explantation), second hospital stay (implantation), and aggregated total hospital stay. The variable costs, which can be more easily influenced by the operating hospital department, account for 22.0 % of the explantation costs versus 58.0 % of the costs associated with the implantation procedure. Unlike the fixed case costs (*p* = 0.214), the variable case costs were significantly different between explantation and implantation (*p* < .001).

#### Reimbursement and contribution margin

Total reimbursement was €24,201.4 per patient. Of the 30 explantations performed, 25 (83.3 %) were assigned to DRG I03 (revision or replacement of the hip joint with complicated diagnosis or arthrodesis or age < 16 with (I03A) or without (I03B) extremely severe complications and/or comorbidities), while 29 implantations were assigned to this DRG (96.7 %) (I03A: *n* =14, 46.7 %, I03B: *n* = 15, 50.0 %). Thus, the data set is quite homogenous. Compared with the mean hospital stay in Germany for I03A (26.7 days), the patients of our study had shorter hospital stays (explantation: 22.1 days, implantation: 21.9 days). However, this does not hold for the subset of less severe I03B cases (mean hospital stay in Germany: 17.8 days; study: explantation: 18.0 days, implantation: 17.4 days) [20]. The average revenues generated by the hospital were €11,922.7 for an explantation and €12,278.7 for an implantation (*p* = .641).

Table 4 shows contribution margins separately for explantations and implantations and aggregated hospital stays.

**Table 4:** Marginal contributions of septic explantations and implantations [€] (range)

	Explantation (*n* = 30)	Implantation (*n* = 30)	Complete case^a^ (*n* = 30)
Cost	4147.4 (1941–12,192.3)	6680.7 (3932.8–13,661.8)	10,828.1 (5083.6–21,875.7)
DRG Revenue	11,922.7 (6797.8–14,610.3)	12,278.7 (9817.7–16,552.1)	24,201.4 (16,615.5–31,162.4)
- Variable Costs	910.6 (253.6–4741.5)	3876.3 (1583.1–8568.0)	4786.9 (2176.8–13,309.5)
= Marginal Contribution I	11,012.1 (6478.8–14,244.6)	8402.4 (4393.5–13,027.2)	19,414.4 (13,472.8–25,746.0)
- Case-Fixed Costs	3236.8 (1583.2–9083.9)	2804.4 (1005.8–6726.1)	6041.2 (2589.1–13,773.2)
= Marginal Contribution II	7775.3 (2418.0–12,622.4)	5598.0 (948.5–10,534.5)	13,773.3 (5348.5–2,3156.9)
ᅟ			
Marginal Contribution I/day	586.8 (282.0–1092.74)	451.0 (191.0–1091.8)	500.8 (284.1–1029.8)
Marginal Contribution II/day	429.3 (69.1–971.0)	305.5 (43.1–877.9)	353.2 (131.2–926.3)

^a^Complete case is the aggregate of the two separate hospital stays for explantation and implantation

*DRG* diagnosis related groups

Both contribution margins, I (*p* = 0.001) and II (*p* = 0.004), differ significantly between explantation and implantation. In each single case, both variable and fixed case costs were covered by revenues. Our analysis yielded a marginal contribution II per day of €429.3 for explantations and of €305.5 for implantations; the difference is significant (*p* = 0.019).

## Discussion

To our knowledge, this is the first study presenting a detailed analysis of the cost of septic revision THA. Unlike previously published studies, our approach does not exclusively rely on reimbursement data but instead breaks down the costs incurred by an orthopedic department in performing revision THA separately for explantation and implantation.

Most investigators report overall cost of septic revision THA [10–12, 21–25], which precludes direct comparison with our results as we did not include the hospital’s fixed costs. An analysis of the fixed costs of a hospital relies on a number of assumptions, and these costs are of limited value for decision making and planning from the individual department’s perspective. For example, a total cost calculation based on data of the preceding year yields accurate results only for the time point of identical total case numbers, all other things being equal, while costs are underestimated before and overestimated after that date. Moreover, the hospital’s fixed costs are more rigid and can only be influenced on a longer-term basis. This means that they can be neglected in an initial analysis of processes aimed at identifying cost factors that are relevant for decision-making from the point of view of the department providing the services.

As the clinical picture which leads to septic THA revision is dangerous and often life-threatening, decisions are driven by medical needs rather than economic considerations. Surprisingly, our analysis revealed that the department’s total cost for a septic revision THA procedure was €10,828.1 - far below the average reimbursement of €24,201.4. Thus, a considerable proportion of the average reimbursement for septic THA revision (55.3 %) contributes towards the hospital’s fixed costs, which are not included in our analysis. Published data suggest that the average fixed costs of a hospital account for 65–75 % of its total cost [16, 17]. While our data provide no information on whether the entire hospital makes a profit or loss, they show that the orthopedic department makes no deficit when performing septic revision THA. The largest shares of the variable case costs are accounted for by staff (36.4 %) and the implant (27.3 %). Due to the costs of the implant, which occur only in second hospital stay variable costs are significant higher for the implantation stay.

Never the less in a hospital all clinical departments are co-responsible of the economic viability. So it is also required to have a global perspective to assure the economic sustainability of the whole hospital. Managers and clinical leaders must cooperate this in line. Positive contribution margins I and II are important but not enough, as also contribution margin III and IV needs to be positive to achieve a positive over all result.

While DRG-based reimbursement (€11,922.7 for explantation vs. €12,278.7 for implantation) and hospitalization duration (20.5 days for explantation vs. 19.8 days for implantation) are nearly identical for the two procedures, the department’s costs related to implantation are much higher than for explantation (difference of €2533.3). This difference is mainly attributable to implant prices, which account for a large proportion of the total cost. Hence, we may conclude that cost savings can be realized by negotiating better purchase prices with suppliers. As patients in need of septic revision THA have an above-average morbidity and need more nursing and physician care at the time of explantation surgery, staff costs on the normal ward also differ significantly between explantation and implantation despite similar hospitalization durations (€1361.6 vs. €909.4, *p* < 0.001). It should be pointed out here seriously that, while cutting cost may be essential for the economic survival of many hospitals patient safety and good outcome must always come first. Economic and medical management must work to together to find payable but medically acceptable solutions.

Comparison of daily contribution margins allows us to compare how much individual DRGs can contribute to the payment of a hospital’s fixed costs. In the case analyzed here, contribution margins per day show that the explantation procedure results in a higher financial benefit for the orthopedic department than the implantation procedure (€429.3 versus €305.5). Although patients are very sick at the time of explantation, the higher contribution margin might be attributable to the fact that no implant costs are involved during the first stage of septic THA revision. With regard to previously published data septic implantations seem to be comparable to aseptic THA revisions (€298.2) in terms of contribution margins [26]. This is very surprising because septic procedures are more difficult and complex. Overall, our analysis shows that both explantation and implantation have a large share in paying the fixed costs of a hospital.

### Limitations

Our study has some limitations, which must be taken into account when interpreting our results. The retrospective nature has the usual advantages and disadvantages of this design in terms of data quality, number of patients included, and representativeness. The total number of cases analyzed is not large, but this ensures that consistent data were available for all patients included. Due to technical aspects it was not possible to calculate costs for staff from other medical disciplines, e.g., during councils, thus the real costs for the hospital stay are slightly underestimated. As we present data from Germany, the results has to be interpreted and compared with regard to the diverging international health care systems and economic circumstances. Nevertheless our approach to calculate contribution margins is transferable. Compared with published data, the operation time and length of hospital stays appear rather short in our patients, which might suggest that the patients of our study population were in less serious conditions and thus could be treated at lower costs. Comparison with future studies will show whether our population is representative.

## Conclusions

Our analysis for the first time provides orthopedic surgeons with data on those parts of hospital costs they can influence. It might also assist hospitals in scenario planning with regard to the overall economic context. In summary, our cost analysis of septic revision THA procedures in two sessions indicates that more than half of the per-case reimbursement (55.3 %) is available for covering the hospital’s fixed costs. Hence, our hypothesis that hospital departments incur an unreimbursed loss when performing septic revision THA has been refuted. None of the 30 cases included in our analysis produced a loss for the orthopedic department. Our detailed analysis of explantation and implantation procedures for the first time shows that variable costs account for a large proportion of the costs associated with implantation procedures and might be a promising target for cost-saving measures. Purchase prices for hip implants and staff cost have been revealed as major cost factors that the hospital might influence.

## Abbreviations

CM: contribution margin
DRG: diagnosis related groups
ICU: intensive-care-unit
THA: total hip arthroplasty
